# Effects and Mechanism of Salvianolic Acid B on the Injury of Human Renal Tubular Epithelial Cells Induced by Iopromide

**DOI:** 10.3389/fphar.2021.761908

**Published:** 2021-12-31

**Authors:** Shu-Jun Dong, Xin-Yue Gao, Ming-Xin Pei, Ting Luo, Dong Fan, Yan-Ling Chen, Jun-Feng Jin, Xiao-Duo Zhao

**Affiliations:** ^1^ Department of Pathophysiology, Zhuhai Campus of Zunyi Medical University, Zhuhai, China; ^2^ Department of Pathology, Suining Central Hospital, Suining, China; ^3^ Department of Pathology, Zhuhai Campus of Zunyi Medical University, Zhuhai, China; ^4^ Zhongshan School of Medicine, Sun Yat-Sen University, Guangzhou, China

**Keywords:** salvianolic acid B, iopromide, contrast-induced nephropathy, endoplasmic reticulum stress, HK-2 cell

## Abstract

With the increasing application of medical imaging contrast materials, contrast-induced nephropathy (CIN) has become the third major cause of iatrogenic renal insufficiency. CIN is defined as an absolute increase in serum creatinine levels of at least 0.50 mg/dl or an increase >25% of serum creatinine from baseline after exposure to contrast. In this study, the protective effects of salvianolic acid B (Sal B) were detected in human renal tubular epithelial cells (HK-2) exposed to iopromide. The results showed that different concentrations of Sal B counteract the loss of cell viability induced by iopromide, and reduce cell apoptosis, the reactive oxygen species (ROS) levels, and the levels of endoplasmic reticulum stress (ERS)–related and apoptosis-related proteins such as p-IRE-1α, p-eIF-2α/eIF-2α, p-JNK, CHOP, Bax/Bcl-2, and cleaved caspase-3. In addition, Sal B at a concentration of 100 μmol/L inhibited ERS and reduced cell damage to a similar extent as the ERS inhibitor 4-PBA. Importantly, treatment with Sal B could abolish the injury induced by ERS agonist tunicamycin, increasing cell viability and the mitochondrial membrane potential, as well as significantly reducing ROS levels and the expression of Bax/Bcl-2, cleaved-caspase-3, GRP78, p-eIF2α, p-JNK, and CHOP. These results suggested that the protective effect of Sal B against HK-2 cell injury induced by iopromide may be related to the inhibition of ERS.

## Introduction

The incidence of contrast-induced nephropathy (CIN) is approximately 10–30% in the general population, while in populations with basic nephropathy and nephrotic syndrome, it can reach up to 55 and 100% (Maccariello, 2016; Luk et al., 2017). CIN has been recognized as the third major cause of acute kidney injury and an important factor in the death of percutaneous coronary intervention (PCI) patients with ischemic heart disease (Palli et al., 2014; Sekiguchi et al., 2018). At present, the clinical prevention and treatment of CIN are still limited to hydration therapy, symptomatic treatment, and antioxidant therapy. Therefore, it is important to seek more effective measures to prevent and treat CIN.

Iopromide, a new nonionic hypotonic contrast agent commonly used in PCI, has a direct toxic effect on renal tubular epithelial cells, such as the vacuolation of proximal tubules, interstitial edema, and renal tubular degeneration (Tan et al., 2017). The cytotoxic mechanisms induced by contrast agents include apoptosis, cell inflammation, and calcium homeostasis damage (Rear et al., 2016; Lambert et al., 2017). The flow resistance in the renal tubules increases after renal tubular epithelial cells are injured, and the renal interstitial pressure can reach up to 50 mmHg. This pressure will significantly reduce the blood flow and glomerular filtration rate of the renal medulla, ultimately leading to renal failure (Maccariello, 2016).

The endoplasmic reticulum (ER) is responsible for protein translocation, folding, and posttranslational modification of secreted or transmembrane proteins. When ER conditions change due to hypoxia, starvation, or metabolic dysfunction, a large number of misfolded proteins accumulate together to prompt the ER to initiate an unfolded protein response (UPR) and ER-related degradation pathways to reduce the accumulation of misfolded proteins. This process is called ER stress (ERS) (Iurlaro and Munoz-Pinedo, 2016). ERS is sensed by three upstream signaling proteins: protein kinase RNA-like ER kinase (PERK), inositol-requiring protein-1 (IRE-1), and activating transcription factor-6 (ATF6) (Baiceanu et al., 2016). When misfolded proteins accumulate in the ER, the 78-kDa glucose-regulated protein (GRP78) is activated and folds the misfolded protein while blocking transcription and translation until homeostasis is restored, thereby inhibiting the cascade of cell death (Sisinni et al., 2019). In addition to the PERK-eIF-2α pathway, the expression of C/EBP homologous protein (CHOP) can also be induced by ATF6 and IRE-1 pathways. TRAF2, a tumor necrosis factor receptor–related factor, can also be activated by activated IRE-1, which then activates the apoptosis signal–regulating kinase (ASK1) and c-Jun N-terminal kinase (JNK) pathways. ASK1 and c-Jun can also promote apoptosis, either with or without the activation of CHOP (Kitamura et al., 2011; Feng et al., 2018).

*Salvia miltiorrhiza* has been widely used in the study of kidney protection. Sal B is one of the main active water-soluble polyphenol compounds isolated from *S. miltiorrhiza*. It has been reported to have various pharmacological effects, such as anti-apoptosis, antioxidant, and anti-inflammatory effects (Ding et al., 2016). In a rat kidney injury model induced by an iodinated contrast agent, Sal B enhanced the production of endogenous antioxidants and reduced kidney injury through activating the anti-oxidative stress pathway (Ma et al., 2017). In addition, recent studies have reported that Sal B also has clear anti-ERS effects. In a study of doxorubicin-induced mouse cardiac dysfunction and cardiomyocyte apoptosis, the application of Sal B reduced cardiomyocyte apoptosis by inhibiting ERS (Chen et al., 2016).

There have been many studies on ERS in recent years, but the pathogenesis of ERS in contrast-induced renal injury has not been reported in the literature. Therefore, this work is based on the CIN model to evaluate the protective effect and mechanism of Sal B on HK-2 cells damaged by iopromide to provide an experimental basis for the prevention and treatment of CIN.

## Materials and Methods

### Cell Culture

Human proximal tubular epithelial cells (HK-2; American Type Culture Collection, Rockville, MD, United States) were provided by Professor Weidong Wang, Sun Yat-sen University. The cells were grown in a cell incubator at 37°C in a 5% CO_2_ atmosphere in DMEM/F12 medium supplemented with 10% fetal bovine serum and 1% antibiotics (Gibco, United States).

### Cell Treatments

In order to determine the toxicity of iopromide (BAYER, Germany), HK-2 cells were treated with different concentrations of iopromide (50, 100, 150, and 200 mgI/mL) for 3 h. According to the results, an *in vitro* model of CIN was established using iopromide at 150 mgI/mL.

In order to evaluate the protective effect of Sal B (Ronghe, Shanghai), HK-2 cells were pretreated with different concentrations of Sal B (10, 50, and 100 μmol/L) for 15 min based on the CIN model. To investigate the role of ERS in iopromide-induced injury in HK-2 cells, the ERS inhibitor 4-phenylbutyric acid (4-PBA; MedChemExpress, United States) and the agonist tunicamycin (TM; MedChemExpress, United States) were respectively co-treated with iopromide or Sal B. (Gao et al., 2019).

### Cell Viability Assay

Cell viability was evaluated by the MTT assay. HK-2 cells were cultured to 90% confluency in 96-well plates and received indicated treatments for 3 h. At the end of the indicated treatments, the cells were incubated with serum-free medium containing 10% MTT at 37°C in a 5% CO_2_ atmosphere for 3 h, and then 150 μL DMSO was added to dissolve the formazan crystals in each well. Absorbance was evaluated using a microplate reader (Thermo Fisher Scientific, United States) at a wavelength of 490 nm. Viability was calculated using the formula: cell proliferation rate (%) = OD treatment group/OD control group × 100%. This assay was repeated five times.

### Detection of Nucleus Morphology

HK-2 cells were digested and seeded in 6-well plates, grown to 90% confluency, and received indicated treatments for 3 h. The original medium was aspirated, cells were incubated with 4′,6-diamidino-2-phenylindole (DAPI) staining solution (1 μg/L) in an incubator for 15 min. The cells were washed with phosphate-buffered saline (PBS) three times and visualized under a fluorescence microscope (OLYMPUS, Japan) at ×400 magnification. The experiment was repeated three times.

### Examination of Mitochondrial Membrane Potential

The HK-2 cells were cultured in 6-well plates at 37°C in a 5% CO_2_ atmosphere and collected for determining the mitochondrial membrane potential (MMP). The cells were washed three times with PBS. Then, Rh123 solution (100 μg/L) in serum-free DMEM was added to the plates, which were incubated at 37°C for 15 min. The cells were washed three times with PBS, then imaged under a fluorescent microscope at ×200 magnification and analyzed using ImageJ software.

The cells were cultured in 6.0-cm petri dishes, given the indicated treatment for 3 h, and then trypsin without EDTA was added to detach the adherent cells. The cells were subsequently incubated with 100 μg/L Rh123 at 37°C for 15 min and then washed with PBS three times. Fluorescence was detected by flow cytometry (Partec, Germany) after resuspending the cells in PBS. The experiment was repeated three times and analyzed with FCS Express software (version 4.0).

### Measurement of Intracellular Reactive Oxygen Species Generation

The HK-2 cells were cultured in 6-well plates at 37°C in a humidified atmosphere with 5% CO_2._ Following treatment for 3 h, cells were collected to determine intracellular ROS generation. The cells were washed three times with PBS, then DCFH-DA solution (1 μg/L) in serum-free DMEM was added to the plates, and cells were incubated at 37°C for 15 min. The cells were washed three times with PBS, and then imaged using a fluorescence microscope at ×200 magnification and analyzed using ImageJ software.

The cells were cultured in 6.0-cm petri dishes, given the indicated treatment for 3 h, and trypsin without EDTA was added to detach the adherent cells. The cells were subsequently incubated with 1 μg/L DCFH-DA at 37°C for 15 min and then washed with PBS three times. Fluorescence was detected by flow cytometry (Partec, Germany) after resuspending the cells with PBS. The experiment was repeated three times and analyzed with FCS Express software (version 4.0).

### Detection of Cell Apoptosis

HK-2 cells were cultured in 6.0-cm petri dishes at a density of 4.0×10^6^ cells/mL. The cells were incubated at 37°C until they reached 90% confluency, when they were given their indicated treatments for 3 h. The cells were washed three times with PBS and then digested by trypsin without EDTA. The original culture medium, the washing solution, and the cells were collected in 15-ml centrifuge tubes and centrifuged. Then 500 μL buffer was added to resuspend the cells. The cells were treated with 5 μL of FITC-Annexin V at 37°C and 5% CO_2_ for 10 min. Then 5 μL of propidium iodide was added to the mixture, and the cells were stained for 5 min in an incubator. Finally, the cells were resuspended with 3 mL PBS, detected using flow cytometry, and analyzed with FCS Express software (version 4.0). The experiment was repeated three times.

### Protein Extraction and Western Blot Analysis

Following treatment, the HK-2 cells were lysed with RIPA lysis buffer for 30 min at 4°C. The total protein was quantified using a BCA protein assay kit and separated by 8, 10, or 12% SDS-PAGE. The separated proteins were transferred onto a polyvinylidene fluoride membrane and blocked with 5% fat-free milk for 2 h at room temperature. The membranes were then incubated with the following primary antibodies at 4°C overnight: GRP78 (1:1000, CST, United States), p-IRE-1α (1:1000, CST, United States), p-eIF-2α (1:1000, CST, United States), eIF-2α (1:1000, CST, United States), p-JNK (1:1000, CST, United States), JNK (1:1000, CST, United States), CHOP (1:1000, CST, United States), Bax (1:1000, CST, United States), Bcl-2 (1:1000, CST, United States), and cleaved caspase-3 (1:1000, CST, United States). Following incubation with the primary antibody, the membranes were washed with PBS containing 0.1% Tween-20 and then incubated with the secondary antibody (1:5000, CST, United States) for 1 h at room temperature. The membranes were washed with 0.1% PBS Tween-20, and protein bands were visualized using ECL reagents (Millipore, United States) and exposed to an infrared laser scan-imaging instrument (Analytik Jena, Germany). To semi-quantify protein expression levels, the protein bands were scanned and analyzed for densitometry using ImageJ software. The experiment was repeated three times.

### Statistical Analysis

All data are presented as the mean ± SD. The statistically significant differences between the multiple groups were determined by one-way analysis of variance (ANOVA) followed by the Fisher's least significant difference (LSD) post-hoc test using SPSS 20.0 software. Values of *P* < 0.05 were considered statistically significant.

## Results

### Sal B Counteracted the Decrease of Cell Viability Caused by Iopromide

**FIGURE 1:**
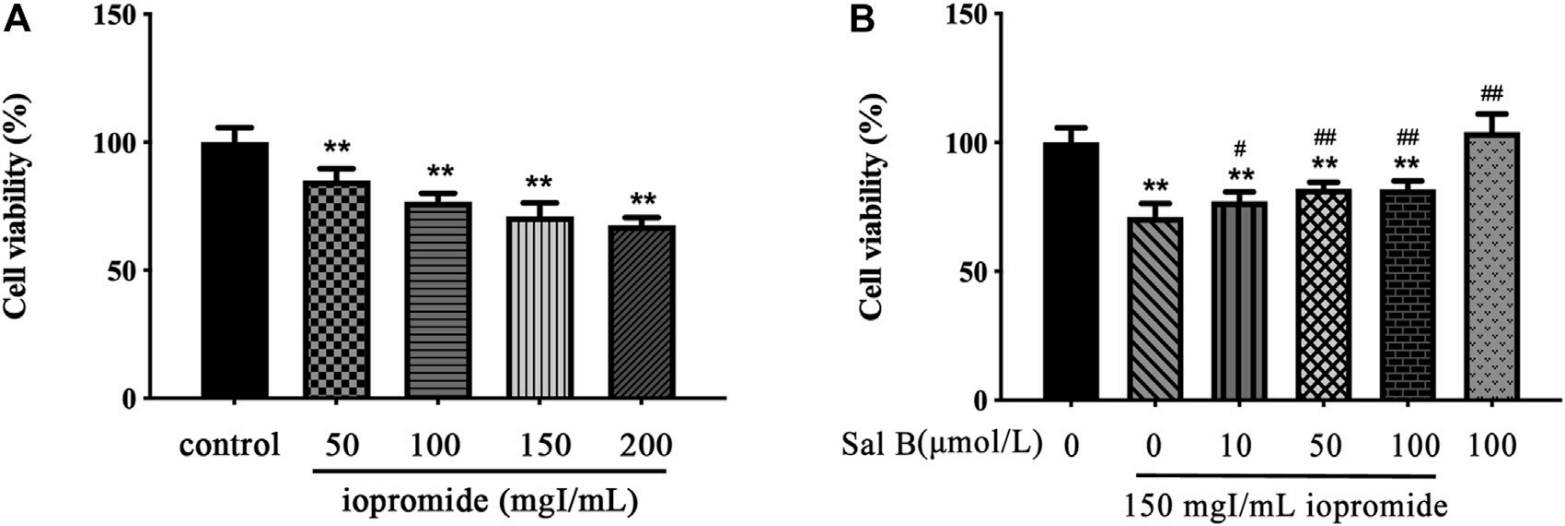
Effect of iopromide and Sal B on cell viability in HK-2 cells. **(A)** Different doses of iopromide were found to cause a significant decrease in cell viability. **(B)** Different doses of Sal B protected HK-2 cells against iopromide-induced injury. The data were determined by ANOVA and LSD post-hoc test and are presented as the mean ± SD (*n* = 5). ^**^ *P* < 0.01 vs. control group; ^#^ *P* < 0.05, ^##^ *P* < 0.01 vs. 150 mgl/mL iopromide group.

HK-2 cells were treated with four concentrations of iopromide (50, 100, 150, and 200 mgI/mL) for 3 h. We observed a dose-dependent decrease in cell viability (Figure 1A). For this reason, we decided to perform subsequent experiments with 150 mgI/mL iopromide. To test whether Sal B could counteract the loss of cell viability caused by iopromide, we treated HK-2 cells with three different concentrations of Sal B (10, 50, and 100 μmol/L) (Figure 1B). Cell viability was significantly increased, indicating that Sal B can counteract HK-2 cells' injury caused by iopromide.

### Effect of Sal B on Iopromide-Induced Changes in GRP78, p-IRE-1α, p-JNK, p-eIF-2α, CHOP, Bax/Bcl-2 Ratio, and Cleaved Caspase-3 Protein Levels in HK-2 cells

**FIGURE 2:**
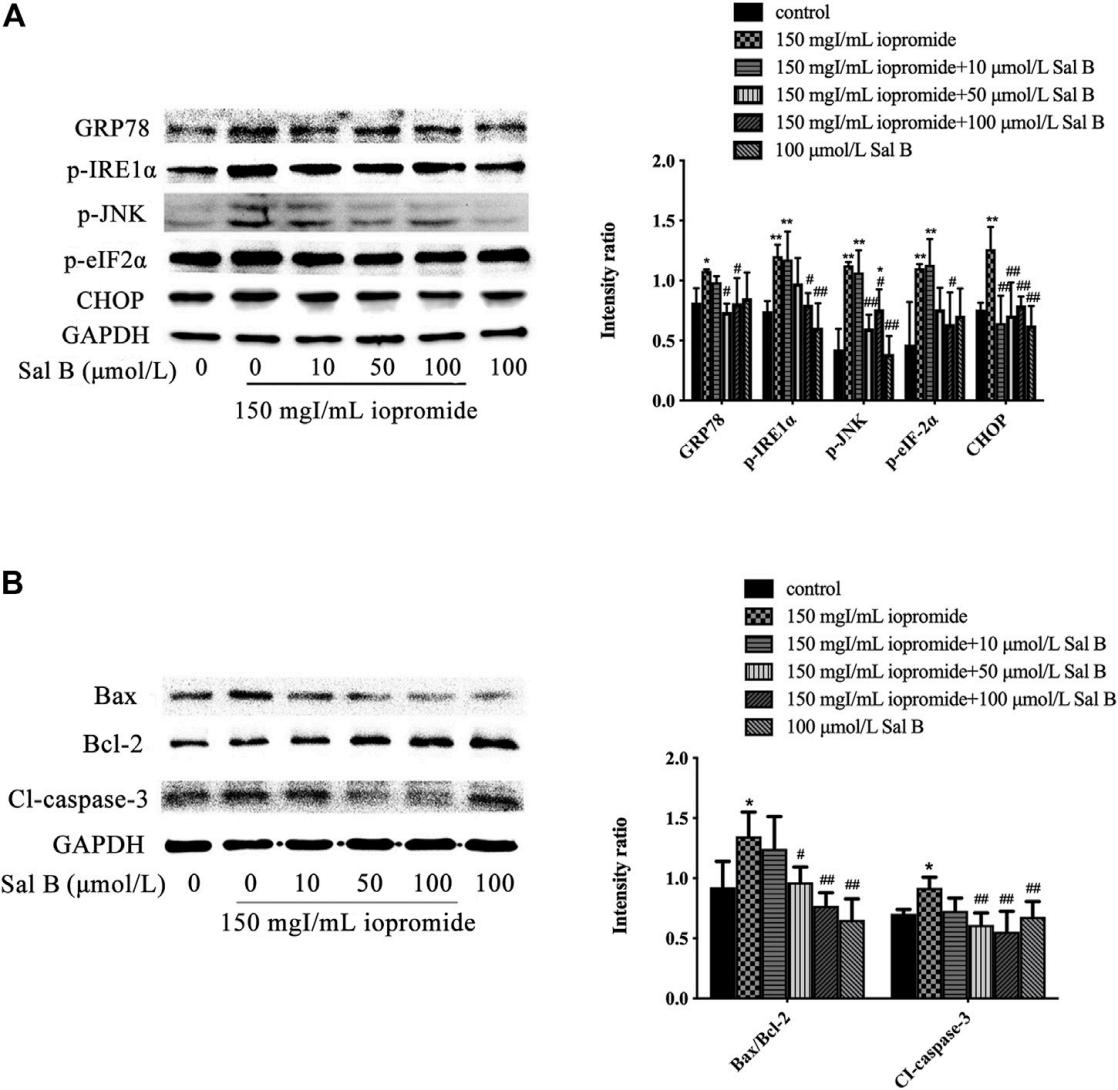
Different concentrations of Sal B were examined for effects on iopromide-induced GRP78, p-IRE-1α, p-JNK, p-eIF-2α, CHOP, Bax/Bcl-2, and cleaved caspase-3 protein levels in HK-2 cells. **(A)** Iopromide treatment caused a significant increase in GRP78, p-IRE-1α, p-JNK, p-eIF-2α, and CHOP. Sal B counteracted the iopromide-induced increase in these protein levels. **(B)** The protein levels of Bax/Bcl-2 and cleaved caspase-3 were significantly increased in iopromide-treated cells. These changes were alleviated by all tested concentrations of Sal B except for 10 μmol/L. The data were determined by ANOVA and LSD post-hoc test and are presented as the mean ± SD (*n* = 3). ^*^ *P* < 0.05, ^**^ *P* < 0.01 vs. control group; ^#^ *P* < 0.05, ^##^ *P* < 0.01 vs. iopromide group.

Compared with the control group, we found an increase in the expression of GRP78, p-IRE-1α, p-JNK, p-eIF-2α, and CHOP after treatment with iopromide. The protein expressions of GRP78, p-IRE-1α, p-JNK, p-eIF-2α, and CHOP were decreased by Sal B (100 μmol/L) (Figure 2A). Iopromide increased the ratio of Bax/Bcl-2 and the level of cleaved caspase-3. We found that 50 and 100 μmol/L of Sal B reduced the ratio of Bax/Bcl-2 and the expression of cleaved caspase-3 caused by iopromide, but 10 μmol/L of Sal B could not induce these effects (Figure 2B). Sal B or 4-PBA protected HK-2 cells against iopromide-induced decrease in cell viability and ROS generation.

**FIGURE 3:**
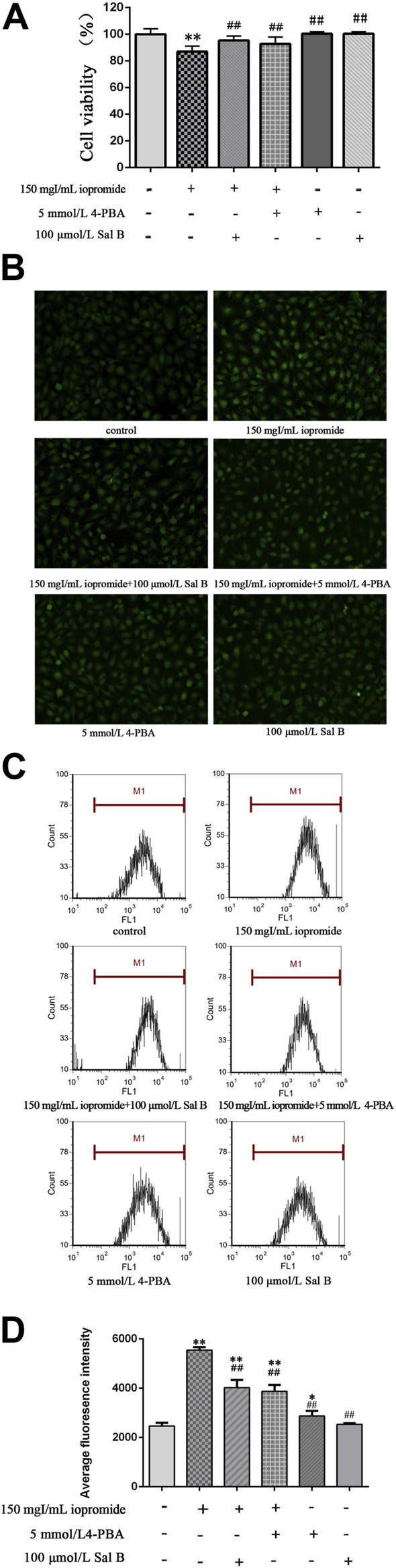
Effect of Sal B or 4-PBA in iopromide-induced decrease in cell viability and ROS generation. **(A)** Compared with the iopromide-treated group, Sal B or 4-PBA alleviated the decrease in cell viability caused by iopromide. The data were determined by ANOVA and LSD post-hoc test and are presented as the mean ± SD (n = 5). ^**^ *P* < 0.01 vs. control group; ^##^ *P* < 0.01 vs. Iopromide group. **(B)** The green fluorescence (readout of ROS level) in the iopromide-treated cells was higher than in the control cells. However, Sal B or 4-PBA could decrease the fluorescence which indicated that they played a protective role by reducing the production of ROS. **(C, D)** Similar results were obtained by flow cytometry. The data were determined by ANOVA and LSD post-hoc test and are presented as the mean ± SD (*n* = 3). ^*^ *P* < 0.05, ^**^ *P* < 0.01 vs. control group; ^##^ *P* < 0.01 vs. iopromide group.

To test whether the protective role of Sal B is exerted through ERS, we treated cells with an ERS inhibitor. We found that cell viability was increased after adding the ERS inhibitor 4-PBA compared with cells treated with iopromide. Importantly, the effect of Sal B on cell viability was similar to that of 4-PBA (Figure 3A). Intracellular ROS generation was measured by determining the oxidation of DCFH-DA. DCFH-DA can penetrate the cell membrane and is hydrolyzed into DCFH by intracellular esterases. The fluorescent probe, which is proportional to the ROS level accumulates and shows green fluorescence. Compared with the control group, the fluorescence intensity of the iopromide group was significantly increased, but administration of Sal B or 4-PBA resulted in a lower level of green fluorescence (Figure 3B). Similar results were obtained by flow cytometry. Cells treated with iopromide showed higher mean fluorescence intensity than the control cells. However, the increase in ROS was abolished by treatment with Sal B or 4-PBA (Figures 3C,D).

### Sal B or 4-PBA Protected HK-2 Cells Against Iopromide-Induced Loss of MMP

**FIGURE 4:**
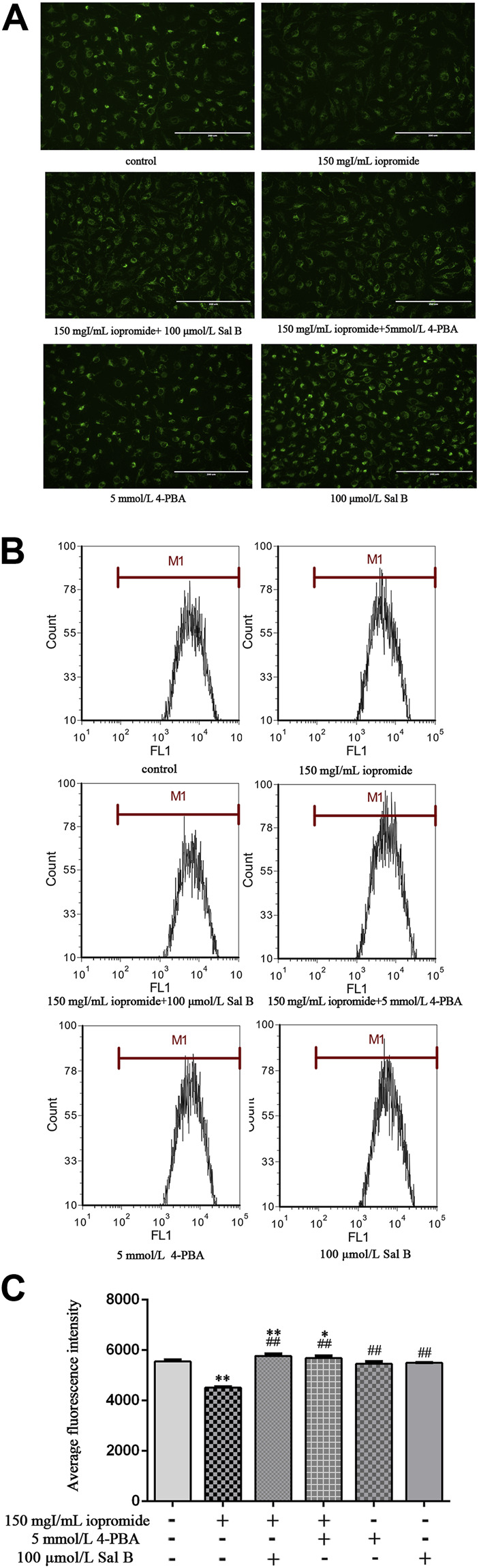
Effect of Sal B or 4-PBA in iopromide-induced loss of the MMP. **(A)** The green fluorescence (readout of MMP) in the iopromide-treated cells was lower than in the control cells. However, Sal B or 4-PBA increased the fluorescence, indicating that they played a protective role by maintaining and/or restoring the MMP. **(B, C)** Similar results were obtained by flow cytometry. The data were determined by ANOVA and LSD post-hoc test and are presented as the mean ± SD (*n* = 3). ^*^ *P* < 0.05, ^**^ *P* < 0.01 vs. control group; ^##^ *P* < 0.01 vs. iopromide group.

The MMP, which is an indicator of mitochondrial function, was analyzed using Rh123. Iopromide promoted a significant loss of the MMP (as measured by a decrease in green fluorescence compared with the control group), indicating that iopromide may induce mitochondrial damage. However, the loss of the MMP and a decrease in green fluorescence were significantly reversed by the treatment with Sal B or 4-PBA (Figure 4A). Iopromide-induced loss of the MMP and the protective effects of Sal B or 4-PBA were also detected by flow cytometry (Figures 4B,C).

### Sal B or 4-PBA Protected HK-2 Cells Against Iopromide-induced Apoptosis

**FIGURE 5:**
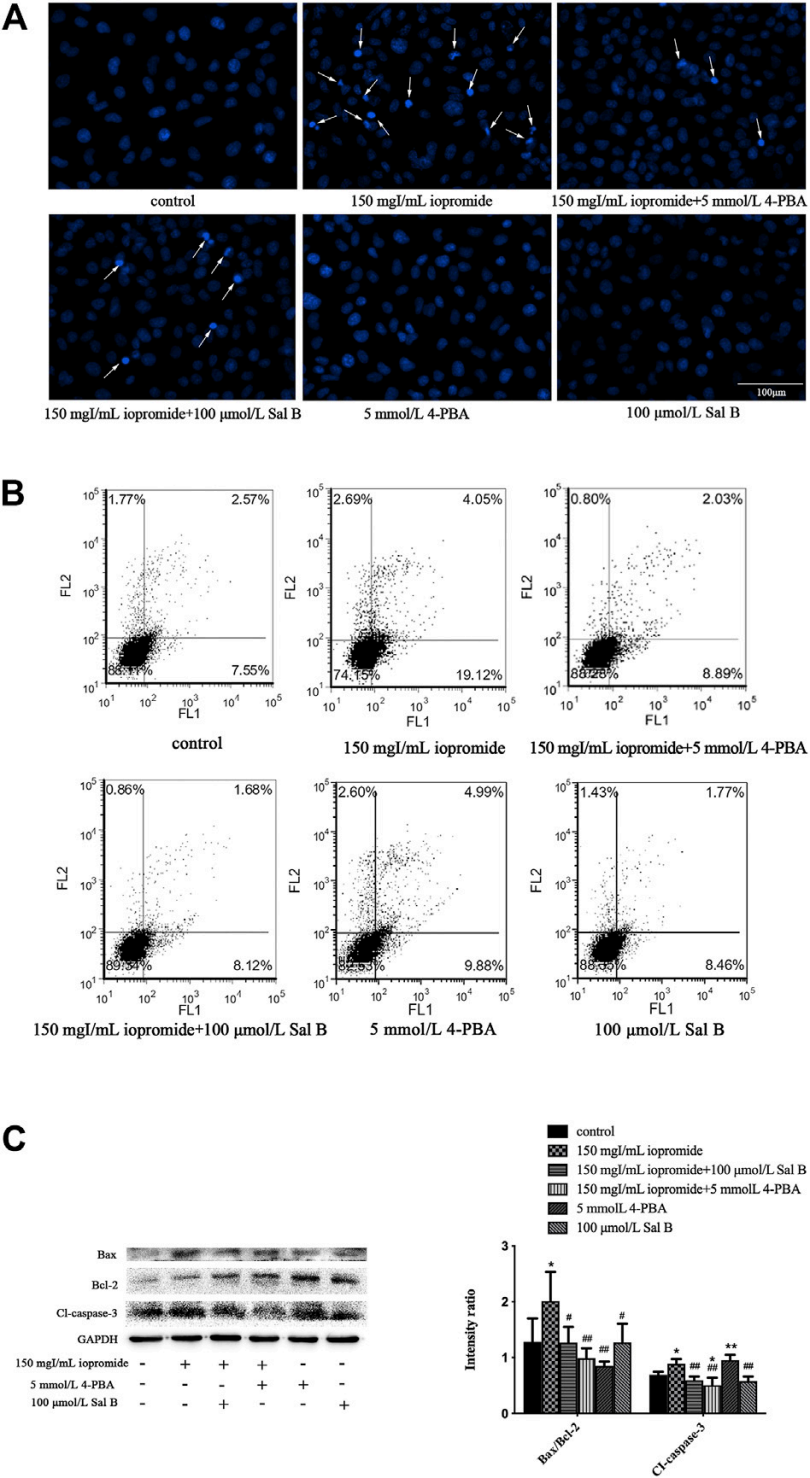
Sal B or 4-PBA counteracted iopromide-induced apoptosis. **(A)** Sal B or 4-PBA attenuated the iopromide-induced apoptosis of HK-2 cells. **(B)** Sal B or 4-PBA reduced the number of apoptotic cells. **(C)** Sal B or 4-PBA reduced the ratio of Bax/Bcl-2 and level of cleaved caspase-3. The data were determined by ANOVA and LSD post-hoc test and are presented as the mean ± SD (*n* = 3). ^*^ *P* < 0.05, ^**^ *P* < 0.01 vs. control group; ^#^ *P* < 0.05, ^##^ *P* < 0.01 vs. iopromide group.

The morphology of the nuclei was observed by DAPI staining. The nuclei of the control, 4-PBA, and Sal B groups were uniformly stained and showed low-density blue fluorescence. After 3 h of treatment with iopromide, a part of the nuclei showed high-density fluorescence and apoptotic characteristics such as karyopyknosis and karyorrhexis. Treatment with Sal B or 4-PBA reduced these indicators of apoptosis (Figure 5A). The increased number of apoptotic cells was verified by flow cytometry (Figure 5B). Iopromide significantly increased the ratio of Bax/Bcl-2 and the level of cleaved caspase-3. Treatment with either Sal B or 4-PBA could reverse these increases to a similar extent (Figure 5C).

### Effect of Sal B or 4-PBA on the Expression of Endoplasmic Reticulum Stress–Related Proteins, Including CHOP, GRP78, p-JNK, JNK, p-eIF-2α, eIF-2α, and p-IRE-1α

**FIGURE 6:**
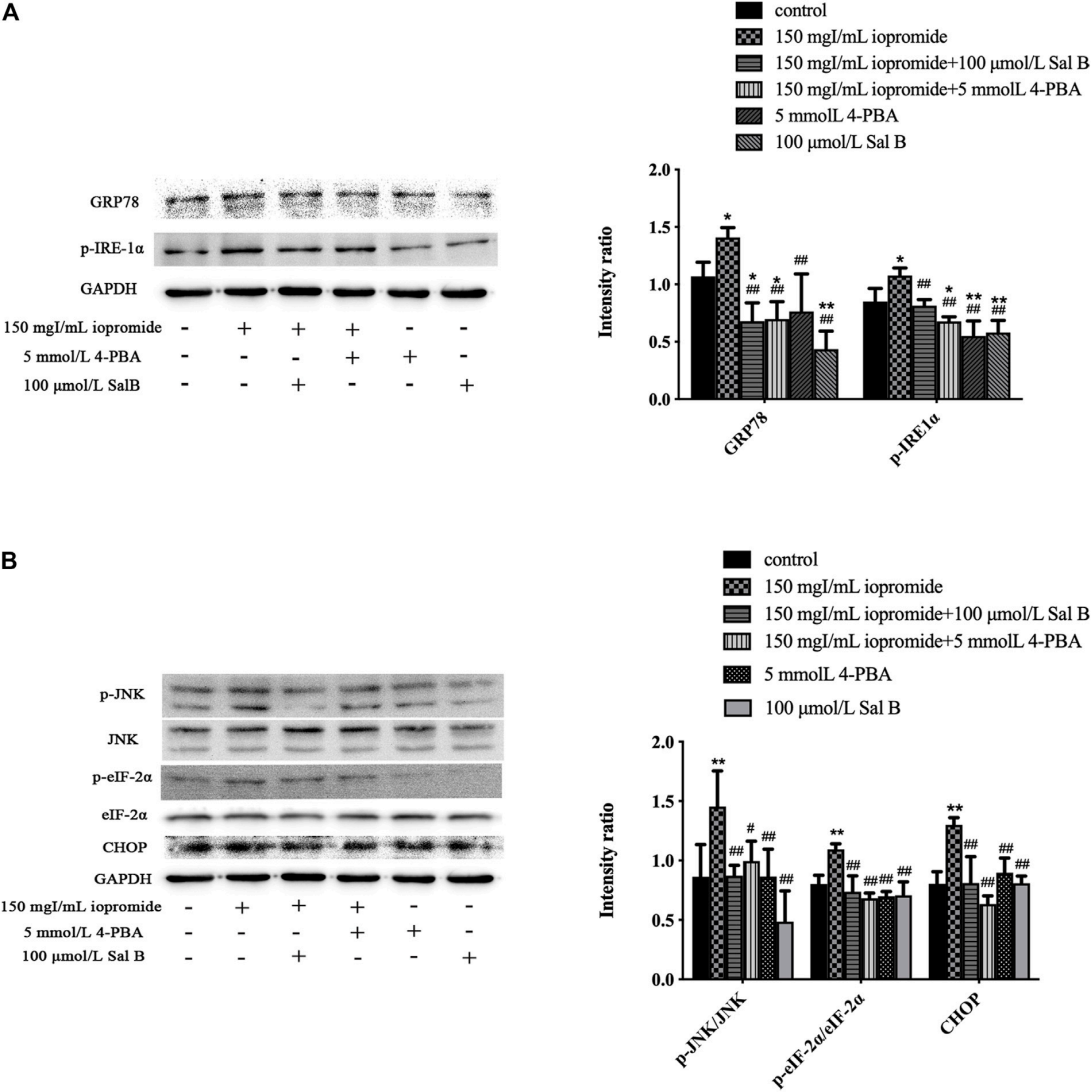
Sal B or 4-PBA significantly reduced the level of ERS-related proteins induced by iopromide. **(A)** Sal B or 4-PBA reduced the level of GRP78 and p-IRE-1α. **(B)** Sal B or 4-PBA decreased the levels of p-JNK/JNK, p-eIF-2α/eIF-2α, and CHOP. The data were determined by ANOVA and LSD post-hoc test and are presented as the mean ± SD (*n* = 3). ^*^ *P* < 0.05, ^**^ *P* < 0.01 vs. control group; ^#^ *P* < 0.05, ^##^ *P* < 0.01 vs. iopromide group.

Treating cells with 150 mgI/mL iopromide significantly increased the levels of GRP78, p-IRE-1α, p-JNK, JNK, p-eIF-2α, eIF-2α, and CHOP (Figures 6A,B) compared with the control group, as detected by western blotting. We found that treatment with either Sal B or 4-PBA significantly rescued these iopromide-induced changes.

### Sal B Counteracted Decreased Cell Viability and Increased Apoptosis Caused by Tunicamycin

**FIGURE 7:**
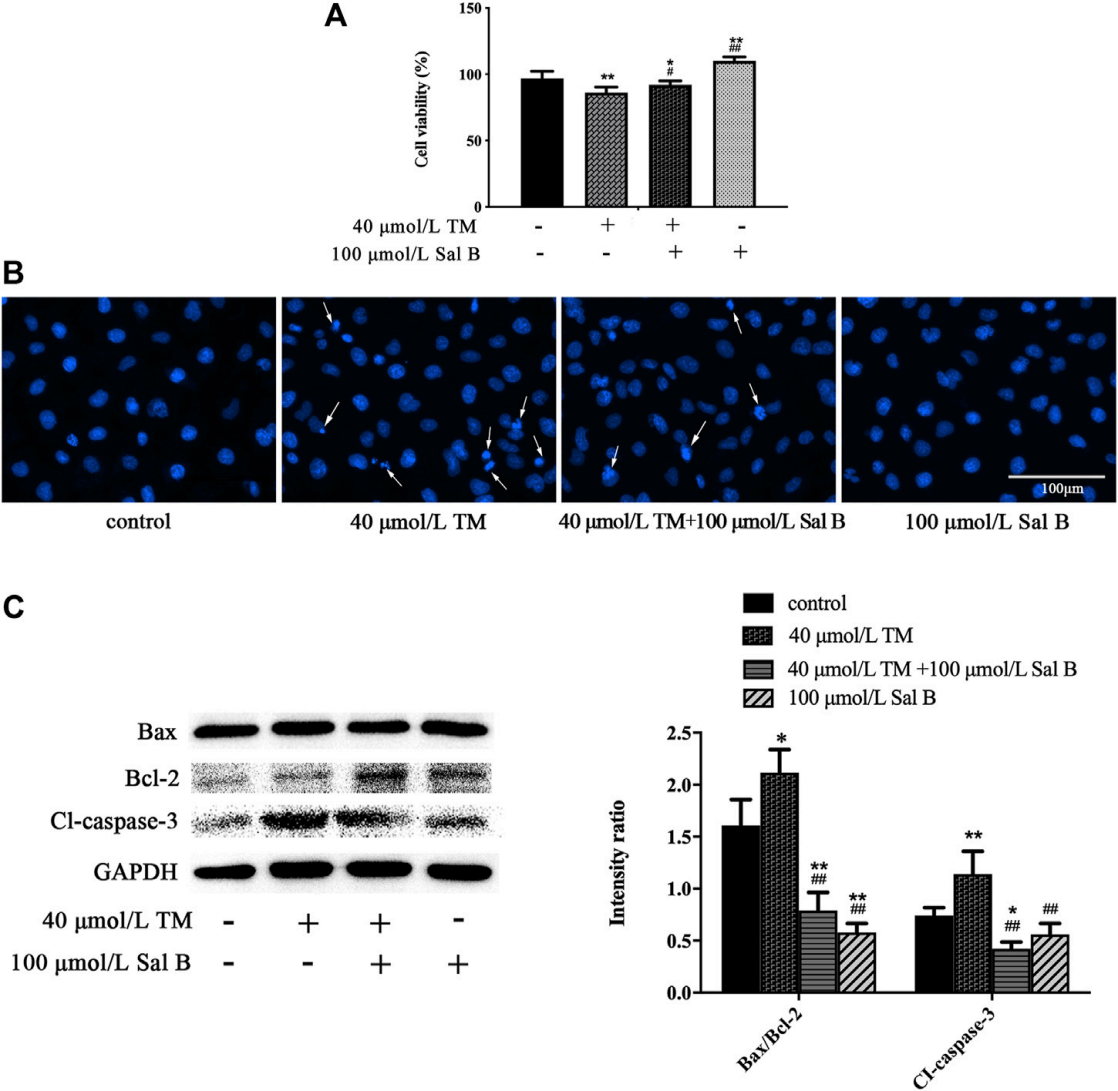
Sal B counteracted TM-induced apoptosis. **(A)** Sal B alleviated the decrease in cell viability caused by TM. The data were determined by ANOVA and LSD post-hoc test and are presented as the mean ± SD (n = 5). ^*^ *P* < 0.05, ^**^ *P* < 0.01 vs. control group; ^#^ *P* < 0.05, ^##^ *P* < 0.01 vs. TM group. **(B)** Sal B attenuated TM-induced apoptosis of HK-2 cells. **(C)** Sal B reduced the ratio of Bax/Bcl-2 and level of cleaved caspase-3. The data were determined by ANOVA and LSD post-hoc test and are presented as the mean ± SD (*n* = 3). ^*^ *P* < 0.05, ^**^ *P* < 0.01 vs. control group. ^#^ *P* < 0.05, ^##^ *P* < 0.01 vs. TM group.

To further examine the correlation between the protective effects of Sal B and ERS-related pathways, we used the ERS agonist TM for subsequent experiments. Treating HK-2 cells with TM reduced cell viability, and Sal B reversed this decrease (Figure 7A). After 3 h of treatment with TM, the cell nuclei showed high-density fluorescence and karyopyknosis, even karyorrhexis. However, Sal B treatment reduced these indicators of apoptosis (Figure 7B). TM significantly increased the ratio of Bax/Bcl-2 and the level of cleaved caspase-3. After treatment with Sal B, the expression of the above proteins was lower than that in the TM group (Figure 7C).

### Sal B Protected HK-2 Cells Against TM-Induced Increase of ROS and Loss of MMP

**FIGURE 8:**
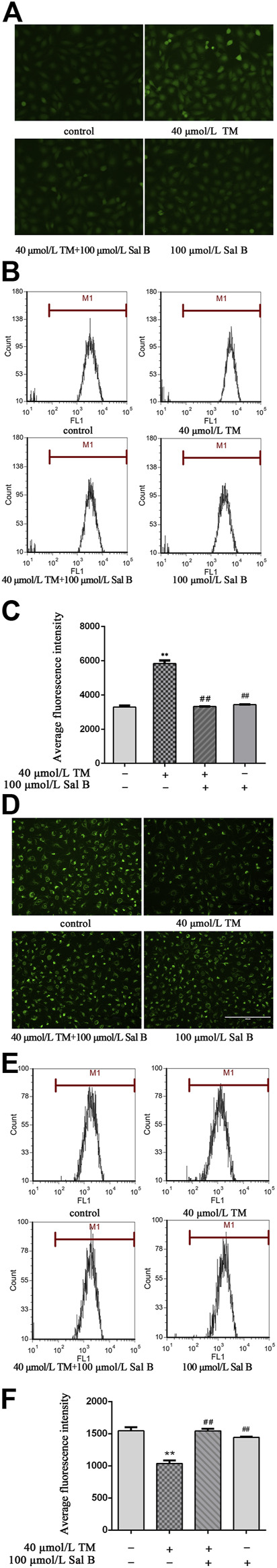
Effect of Sal B on TM-treated cells induced the ROS generation and loss of the MMP. **(A)** The green fluorescence in the TM-treated cells was higher than in the control cells. However, Sal B could reduce the fluorescence intensity. **(B, C)** Similar results were obtained by flow cytometry. **(D)** The green fluorescence in the TM-treated cells was lower than in the control cells. Sal B treatment increased the fluorescence intensity. **(E, F)** Similar results were obtained by flow cytometry. The data were determined by ANOVA and LSD post-hoc test and are presented as the mean ± SD (*n* = 3). ^**^ *P* < 0.01 vs. control group; ^##^ *P* < 0.01 vs. TM group.

TM treatment significantly increased ROS levels, as measured using a fluorescence-based assay. The administration of Sal B reduced the intensity of green fluorescence, indicating that there were lower levels of ROS (Figure 8A). Similar results were obtained using flow cytometry (Figures 8B,C). Additionally, TM induced the loss of the MMP, which was also significantly reversed by the treatment with Sal B (Figure 8D). The TM-induced loss of the MMP and the protective effects of Sal B were also detected by flow cytometry (Figures 8E,F).

### Effect of Sal B on the Expression of Endoplasmic Reticulum Stress–Related Proteins, Including CHOP, GRP78, p-JNK, JNK, p-eIF-2α, eIF-2α, and p-IRE-1α

**FIGURE 9:**
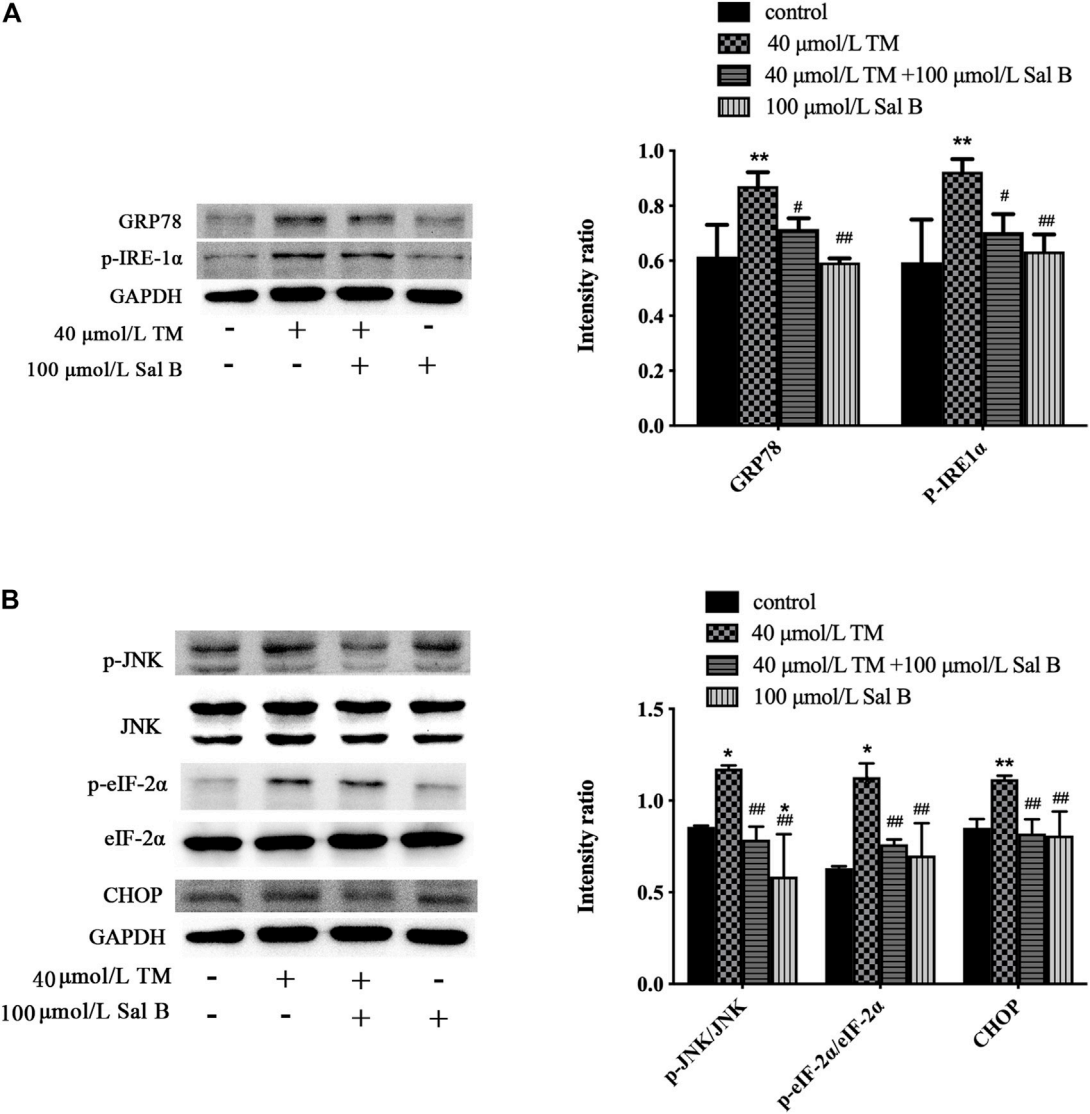
Sal B significantly reduced the level of ERS-related proteins induced by TM. **(A)** Sal B reduced the levels of GRP78 and p-IRE-1α. **(B)** Sal B decreased the levels of p-JNK/JNK, p-eIF-2α/eIF-2α, and CHOP. The data were determined by ANOVA and LSD post-hoc test and are presented as the mean ± SD (*n* = 3). ^*^ *P* < 0.05, ^**^ *P* < 0.01 vs. control group;^#^ *P* < 0.05, ^##^ *P* < 0.01 vs. TM group.

TM significantly increased the levels of the ERS-responsive factors GRP78, p-IRE-1α, p-JNK, JNK, p-eIF-2α, eIF-2α, and CHOP (Figures 9A,B).

## Discussion

**FIGURE 10:**
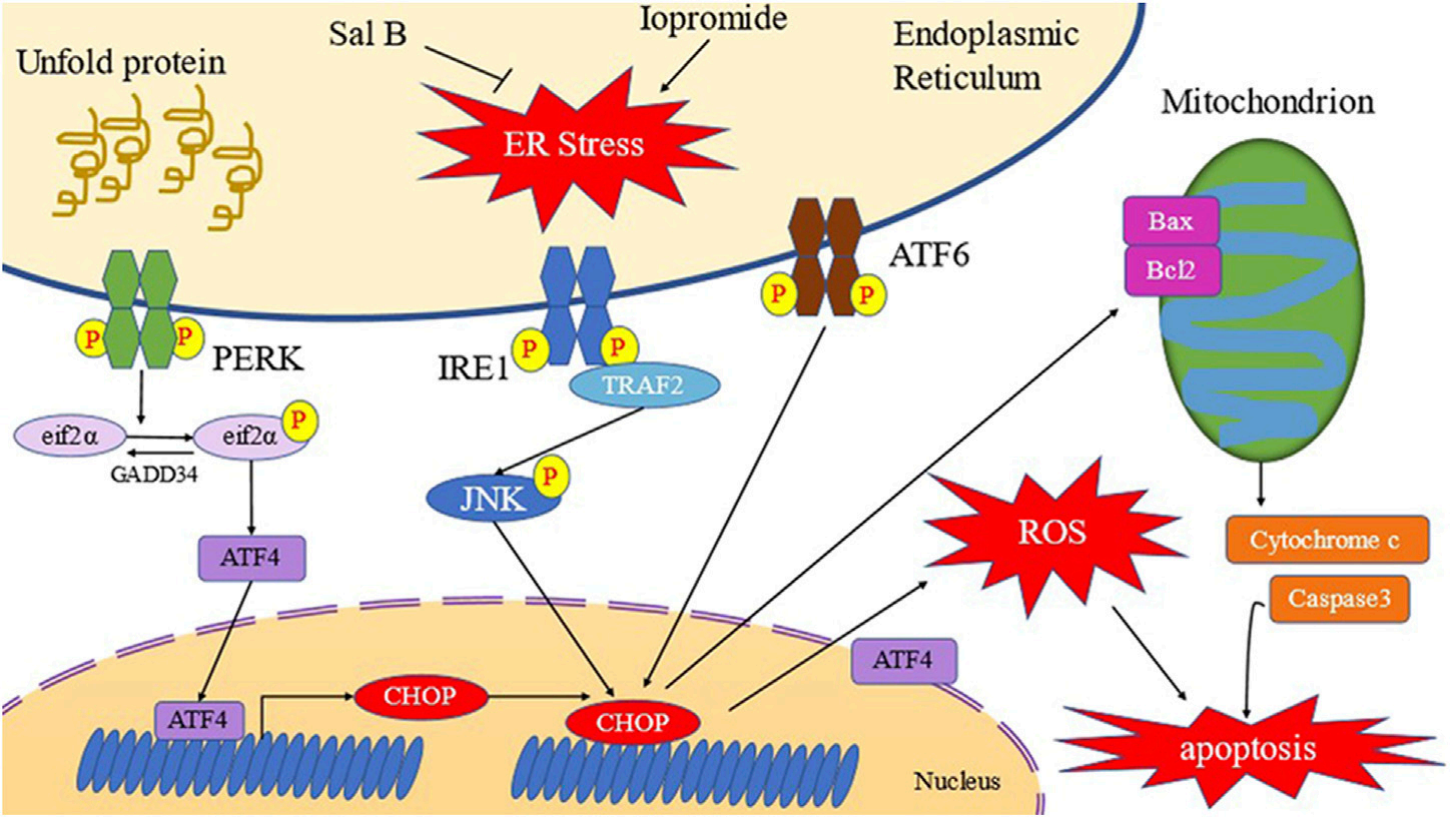
Schematic diagram of Sal B inhibiting ERS of HK-2 cells induced by iopromide (Yang et al., 2020).

Damage to the renal tubular epithelial cells plays a significant role in the occurrence of CIN (Wong et al., 2016; Hossain et al., 2018). Therefore, appropriate measures to reduce the damage to the renal tubules are important for the prevention and treatment of CIN. Studies have reported that iopromide can induce renal tubular epithelial cell damage in *in vitro* experiments (Yang et al., 2018). Therefore, we tested several different concentrations of iopromide (50, 100, 150, and 200 mgI/mL) to treat HK-2 cells for 3 h. Our results show that cell viability of HK-2 cells decreased with an increase in the concentration of iopromide, which is consistent with previous reports. After treatment with Sal B, cell viability of HK-2 cells was significantly increased, indicating that Sal B can resist HK-2 cell damage caused by iopromide (Figure 10).

Sal B is the most abundant and active compound in *S. miltiorrhiza*. Studies have shown that Sal B can reduce cell apoptosis by inhibiting ERS-related proteins such as GRP78 and CHOP (Zhang et al., 2017). Our study shows that Sal B can exert dose-dependent effects on increasing both the levels of ERS-related proteins and cell apoptosis. ERS is driven by signals that communicate in-between the plasma membrane, ER, cytoplasm, mitochondria, and nuclei to alter cell homeostasis, thereby inducing or inhibiting apoptosis (Han and Kaufman, 2016; Ariyasu et al., 2017). Apoptosis eliminates damaged cells in an orderly and effective manner at the gene level (Fuchs and Steller, 2011).

The death receptor pathway is activated by the interaction between the tumor necrosis factor receptor family proteins and their corresponding tumor necrosis factor family ligands (Dickens et al., 2012). The formation of death-inducing signaling complexes activates pro-caspases and leads to the activation of downstream caspase-3, caspase-6, and caspase-7, inducing cell death (Larsen and Sorensen, 2017).

The mitochondrial pathway is mediated by intracellular signals under different stress conditions, such as the lack of oxygen, high concentration of calcium ions, and serious internal stimulation (Pistritto et al., 2016). Subsequently, the antiapoptotic protein B-cell lymphoma 2 (Bcl-2) and the proapoptotic protein Bax act as an “apoptotic switch” by regulating the function of the mitochondrial membranes (Kalkavan and Green, 2018). When Bax is overexpressed, it causes the destruction of the mitochondrial membrane, allowing cytochrome C to diffuse into the cells and plays a key role in activating mitochondria-dependent death in the cytoplasm (Jin et al., 2019). Cytochrome C binds to apoptotic protease activating factor-1 (APAF-1) to form a complex that activates the promoter pro-caspase 9, hydrolyzes the protein, and then activates downstream caspase-3, caspase-6, and caspase-7 (Zhang et al., 2017). Caspase-3, which is thought of as the most critical executor, cleaves actin filaments in a calcium-independent manner. This leads to the destruction of the cytoskeleton, which impairs intracellular transport, cell division, and signal transduction, leading to cell death (Weng et al., 2019). We found that treating HK-2 cells with iopromide resulted in decreased levels of the MMP, and increased both the ratio of Bax/Bcl-2 and the level of cleaved caspase-3, indicating that iopromide induced cell apoptosis through the mitochondrial pathway. Sal B showed dose-dependent rescue of the MMP, Bax/Bcl-2 ratio, and level of cleaved caspase-3, indicating that Sal B had antiapoptotic effects.

The ER provides a unique oxidation environment for proteins to facilitate the formation of disulfide bonds, contributing to approximately 25% of intracellular ROS production (Tu and Weissman, 2004). When the protein translation and folding system is overloaded, ROS production increases in the ER (Ferreiro et al., 2012). The ROS in the ER activates the IP3R1 calcium ion release channel, which results in calcium ions being released into the cytoplasm. Intracellular calcium promotes the production of ROS by activating calcium-dependent protein kinase 2 (CaMKII) and the nicotinamide adenine dinucleotide phosphate (NADPH) oxidase subunit Nox2 on the cell membrane (Li et al., 2009). Kim et al. (2009) reported that the treatment with the ERS agonist TM significantly increased ROS levels in HepG2 cells. Reducing ERS can lead to a decrease in the intracellular ROS levels. Studies of cellular ROS production induced by oxygen and glucose deprivation found that ERS caused an abnormal increase in intracellular ROS by promoting mitochondrial dysfunction and activation of NADPH oxidase (Zeeshan et al., 2016; Ochoa et al., 2018). In addition, the ERS-related PERK and IRE-1 pathways play vital roles in regulating the intracellular ROS levels. The PERK pathway has a negative regulatory effect on the intracellular ROS levels because activated PERK can release nuclear factor E2–related factor 2 (Nrf2) through phosphorylation of Kelch-like ECH-associated protein 1 (KEAP1), which results in Nrf2 translocation into the nucleus to induce expression of antioxidant genes. Conversely, activated IRE-1 promotes intracellular ROS levels, as IRE-1–dependent activation of ASK and P38 MARK contributes to further activation of CHOP production of ROS (Zhong et al., 2015; Wang et al., 2018).

Studies have shown that rotenone (a complex I inhibitor) and Antimycin A complex III inhibitors significantly increased ROS production and induced ERS in mice, while high ROS clearance significantly reduced ERS in mice (Cela et al., 2010). Another study showed that mice fed a methionine/chlorine-deficient diet significantly increased the levels of ROS and ERS-related proteins and that excessive ROS may downregulate the activities of Na^+^/K^+^-ATPase and Ca^2+^-ATPase (Zeeshan et al., 2016), leading to ER injury and the initiation of ERS (Li et al., 2020). In addition, ROS opens the Ca^2+^ channel IP3Rs and ryanodine receptors in the ER, increasing calcium flux into the cytoplasm, which further interferes with protein folding in the ER and causes mitochondrial oxidative stress and dysfunction (Cao and Kaufman, 2014).

The results of this study showed that iopromide increased ROS levels, which can be rescued with an ERS inhibitor. We demonstrated that the effect of Sal B was consistent with that of an ERS inhibitor. We then showed that ROS levels were also significantly increased with the application of an ERS agonist and were decreased with the addition of Sal B, suggesting that the mechanism of Sal B in alleviating the ROS induced by iopromide may be related to the ERS pathway.

ERS-induced apoptosis occurs mainly through three pathways: the IRE-1/ASK1/JNK pathway, the caspase-12 kinase pathway, and the CHOP pathway (Hetz, 2012; Cao et al., 2014). The IRE-1/ASK1/JNK pathway is important in ERS-induced apoptosis and has been demonstrated to be involved in many diseases, such as osteoporosis and urothelial carcinoma (Zeng et al., 2015; Chen et al., 2017). As the core of the UPR, the IRE-1 pathway activates the tumor necrosis factor–related receptors, which then activate the downstream apoptotic signal–regulated kinase 1 (ASK1), JNK, and p38 MAPK pathways (Kim et al., 2018; Yi et al., 2019). The phosphorylation of JNK regulates the expression of Bcl-2 family members, which convey the activation of proapoptotic genes and downregulation of antiapoptotic genes (Lu et al., 2017; Liu et al., 2020). Other studies have shown that in TM-induced apoptosis, the JNK inhibitor SP600125 can inhibit the upregulation of CHOP and the expression of death receptor 5 (DR5), suggesting that the activation of JNK is also involved in the regulation of CHOP (Hotamisligil and Davis, 2016; Guo et al., 2017). In addition, during ERS, PERK promotes the phosphorylation of eIF-2α and enhances the translational activity of ATF4, which in turn induces the upregulation of CHOP (Guo et al., 2020). The PERK-eIF-2α-ATF4-CHOP pathway induces apoptosis by binding to death receptor pathway proteins (Cao et al., 2012; Liu et al., 2016).

CHOP belongs to the CCAAT/enhancer binding protein (C/EBP) family and takes part in the regulation of genes associated with proliferation, differentiation, expression, and energy metabolism. When ERS occurs in cells, the three pathways of PERK, ATF6, and IRE-1 can regulate the CHOP gene (Yang et al., 2017; Wu et al., 2020), which dramatically increases the expression of CHOP protein, activates several upstream proapoptotic signals and acts on the mitochondrial membrane. Activated Bcl-2 protein forms protein channels on the mitochondrial membrane to release apoptosis-activating substances into the cytoplasm (Estaquier et al., 2012). These substances then activate downstream caspase family proteins, which act on their corresponding substrates, leading to cell apoptosis (Iurlaro and Munoz-Pinedo, 2016). As a transcription factor, CHOP can also regulate the expression of many antiapoptotic and proapoptotic genes, such as downregulating the expressions of Bcl-2, Bcl-XL, and Mcl-1 and upregulating the expression of BIM, leading to increased expressions of Bak and Bax (Tsukano et al., 2010; Sano and Reed, 2013). The Bax-Bak complex can release apoptotic factors such as cytochrome C through increased mitochondrial membrane permeability, eventually leading to cell death (Tuzlak et al., 2016). In addition, CHOP also stimulates the expression of proapoptotic proteins by activating DNA damage–inducing protein 34 (GADD34) (Hu et al., 2018).

In our study, the expressions of ERS-related proteins were detected by western blotting; GRP78 is a marker of ERS initiation and plays an important role in maintaining protein stability, regulating protein folding, and inducing apoptosis. Our results showed that iopromide induced an increase in GRP78 levels in HK-2 cells. This indicates that iopromide can induce an ERS response, increase the ratio of Bax/Bcl-2, and decrease the MMP, suggesting that iopromide may induce a decrease in membrane potential through ERS-mediated changes in mitochondrial membrane permeability. In addition, iopromide also increased the expression of cleaved caspase-3 in HK-2 cells, suggesting that iopromide may induce apoptosis in HK-2 cells through the ERS-mediated mitochondrial apoptosis pathway. Treatment with Sal B, reversed the effects that were induced by iopromide, Sal B reduced the level of ERS-promoting protein GRP78 and its downstream factors p-eIF-2α and p-IRE-1α. Moreover, the expression levels of CHOP and JNK were also decreased. This indicated that Sal B may inhibit apoptosis of HK-2 cells induced by iopromide by inhibiting the IRE-1/ASK1/JNK and the PERK-eIF-2α-ATF4-CHOP pathways.

To further explore the role and mechanism of Sal B in ERS caused by iopromide-induced HK-2 cell injury, we introduced the ERS inhibitor 4-PBA and agonist TM to treat the cells. The results showed that 4-PBA not only decreased the expression of ERS-related proteins induced by iopromide but also reduced apoptosis, suggesting that iopromide-induced HK-2 cell injury is closely related to ERS. In addition, we found that the protective effect of Sal B on iopromide-induced HK-2 cell injury was similar to that of 4-PBA. Finally, we found that Sal B can reverse ERS and cell damage induced by TM. In conclusion, we report that Sal B can protect against HK-2 cell injury induced by iopromide by inhibiting ERS.

## Conclusion

ERS is involved in iopromide-induced renal tubular epithelial cell injury. Sal B has a protective effect against iopromide-induced renal tubular epithelial cells injury, and the protective mechanism may be related to the inhibition of ERS.

## Data Availability

The original contributions presented in the study are included in the article/supplementary material, further inquiries can be directed to the corresponding author.
